# Predictive Diagnostic Power of Anthropometric Indicators for Metabolic Syndrome: A Comparative Study in Korean Adults

**DOI:** 10.3390/jcm14020448

**Published:** 2025-01-12

**Authors:** Jongsuk Park, Yonghyun Byun, Sangho Kim

**Affiliations:** 1 School of Global Sport Studies, Korea University, 2511, Sejong-ro, Sejong-si 30019, Republic of Korea; model200@korea.ac.kr; 2 Department of Sports Medicine, Dankook University, 119, Dandae-ro, Dongnam-gu, Cheonan-si 31116, Chungcheongnam-do, Republic of Korea; byunyh@dankook.ac.kr

**Keywords:** metabolic syndrome, anthropometric indicators, predictive power, receiver operating characteristic curve analysis, waist circumference-to-height ratio, body mass index, waist circumference, muscle mass-to-fat mass ratio, muscle mass-to-waist circumference ratio, body shape index, Korean adults

## Abstract

**Background/Objectives:** Metabolic syndrome (MetS) is a cluster of risk factors that significantly increase the risk of cardiovascular disease, including type 2 diabetes, etc. Assessing the predictive diagnostic power of anthropometric indicators for MetS is crucial for the early identification and prevention of related health issues. This study focuses on the Korean adult population while providing insights that may be applicable to broader global contexts. Therefore, this study aimed to compare the predictive diagnostic capabilities of various anthropometric indicators, including body mass index (BMI), waist-to-height ratio (WHtR), muscle mass-to-fat mass ratio (MFR), muscle mass-to-waist ratio (MWR), and body shape index (ABSI), in relation to MetS in Korean adults. **Methods:** Data from 13,725 participants of the Korea National Health and Nutrition Examination Survey (2008–2011) were analyzed. The diagnostic power of each indicator was assessed using the receiver operating characteristic (ROC) curve analysis, and the area under the curve (AUC) values were compared. Participants were classified into normal (NG) and abnormal (AG) groups based on established cutoff values, and logistic regression analysis was performed to evaluate the odds of MetS in each group. **Results:** WHtR showed the highest AUC values (0.792 for men and 0.768 for women), indicating superior diagnostic accuracy compared to the other indicators (*p* < 0.001). Logistic regression analysis indicated that both the unadjusted and adjusted odds ratios (OR) for MetS were significantly higher in the AG than in the NG across all indicators (*p* < 0.001). Specifically, the adjusted OR (95% confidence interval) for WHtR in the AG was 6.793 (5.929–7.784) for men and 4.665 (4.151–5.423) for women, representing the highest values among all indicators (*p* < 0.001). **Conclusion:** Among the various anthropometric indicators, WHtR is the most reliable and practical for predicting MetS in Korean adults. It is useful for early intervention and prevention in both clinical and public health settings.

## 1. Introduction

Metabolic syndrome (MetS) is a medical condition defined by a cluster of metabolic disorders, including hypertension, reduced high-density lipoprotein cholesterol (HDL-C) levels, elevated fasting blood glucose and triglyceride (TG) levels, and excess abdominal fat [1]. The global prevalence of MetS is estimated to range from 12.5% to 31.4%, based on diagnostic criteria [2]. In the Korean adult population, the prevalence of MetS increased from 27.0% in 2007 to 33.2% in 2020 [3]. This rising prevalence highlights the public health importance of MetS as it is strongly associated with an increased risk of cardiovascular and cerebrovascular diseases and higher mortality rates [4,5]. Therefore, effective early detection of MetS is crucial for slowing disease progression and preventing chronic diseases such as diabetes and cardiovascular diseases. The National Cholesterol Education Program Adult Treatment Panel III (NCEP ATPIII) is effective for diagnosing MetS; however, they rely on invasive blood tests and specialized medical evaluations [5]. These requirements limit their practicality for widespread screening and early detection in non-clinical settings. Therefore, there is a growing need for methods that can predict MetS risk earlier and more conveniently. A recent study focused on developing indicators for the early prediction of MetS as part of a prevention and intervention strategy [6].

Receiver operating characteristic (ROC) curve analysis is widely used to assess the accuracy of diagnostic indicators in medicine and the health sciences. Determining the optimal cutoff values for diagnostic tests is crucial for general population screening and diagnosis [7,8]. The area under the ROC curve (AUC) serves as a critical measure of diagnostic power, with a value of 1 indicating perfect accuracy. Sensitivity and specificity are essential metrics for identifying the optimal cutoff values [8].

Recent studies have investigated blood-based biochemical markers such as adiponectin, tumor necrosis factor-alpha, gamma-glutamyl transferase, leptin, uric acid/creatinine ratio, and neutrophil/lymphocyte ratio for predicting and diagnosing MetS due to their metabolic specificity [9,10,11,12,13,14]. However, these markers typically require invasive blood collection and laboratory analyses. Additionally, they are less accessible to public health applications. Therefore, non-invasive indicators, such as body composition and anthropometric indicators, are required to effectively predict MetS.

Regarding non-invasive measurement methods, bioelectrical impedance analysis (BIA) devices are widely used to measure muscle and body fat mass, and tools like measuring tapes can be used to measure waist circumference. Using these indicators, simple formulas can be applied to calculate non-invasive variables such as body mass index (BMI), waist circumference (WC), waist circumference-to-height ratio (WHtR), body shape index (ABSI), waist circumference-to-hip circumference ratio (WHR), muscle mass-to-fat mass ratio (MFR), and muscle mass-to-waist circumference ratio (MWR). These anthropometric measurements are cost-effective and less invasive than blood-based biochemical or imaging-based tests [15]. Previous studies have confirmed the utility of these indicators as effective predictive markers for MetS prevention and management [6,15,16,17,18,19,20,21,22,23].

Despite these advances, some indicators, such as BMI, WHR, ABSI, and WHtR do not fully account for factors such as skeletal muscle mass, which plays a critical role in MetS development [24,25]. Additionally, BMI and MFR overlook the important roles of central obesity and body fat distribution [26,27]. Although several studies have confirmed the predictive power of individual indicators, few have conducted comparative analyses to determine which is the most effective for MetS prediction. To the best of our knowledge, no study has identified the most reliable predictor among the commonly used anthropometric indicators for MetS through direct comparisons of their diagnostic power.

This study aimed to identify the most reliable anthropometric indicator for predicting MetS in its early stages. To achieve this, we evaluated and compared the predictive power of BMI, WHR, WHtR, MFR, MWR, and ABSI for MetS in the Korean adult population. By identifying the most reliable and accessible predictor for MetS this study sought to advance clinical and public health strategies for MetS management, providing a foundation for more efficient and targeted intervention programs.

## 2. Materials and Methods

### 2.1. Data Sources and Study Population

**Figure 1:**
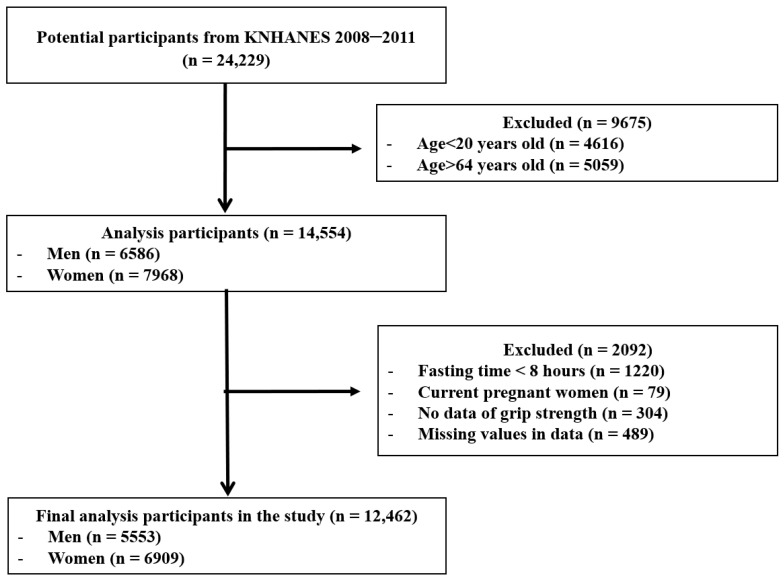
Flowchart for the selection of study participants.

This study used data from the Korea National Health and Nutrition Examination Survey (KNHANES), conducted by the Korea Disease Control and Prevention Agency (KDCA). We performed a secondary analysis using the KNHANES dataset, which includes muscle and body fat mass data collected between 2008 and 2011. This period was selected because it includes measurements of muscle mass and body mass, which were essential variables for evaluating the anthropometric indicator in this study. The initial cohort consisted of 37,753 individuals from the 2008–2011 KNHANES dataset. This study focused on adults aged 19–65 years; therefore, participants aged < 19 years or ≥65 years (*n* = 15,746) were excluded. Additionally, pregnant women (*n* = 157) were excluded to minimize the effects of physiological changes due to pregnancy, and participants who did not fast for at least 8 h (*n* = 1987) were excluded to ensure the accuracy of blood sugar and lipid measurements. Furthermore, participants without muscle and body fat mass measurements (*n* = 5662) and those with missing values for the measurement indices (*n* = 476) were excluded. The final analysis included 13,725 participants (5888 men and 7837 women). All study participants included in the 2008–2011 KNHANES dataset provided their consent to participate in the survey by signing a consent form. The study was conducted in accordance with the Declaration of Helsinki and was approved by the Research Ethics Review Committee of KDCA (approval numbers: 2008-04EXP-01-C, 2009-01CON-03-2C, 2010-02CON-21-C, and 2011-02CON-06-C, respectively). The participant selection process is illustrated in Figure 1.

### 2.2. Definition of Metabolic Syndrome

The diagnostic criteria for MetS in this study were based on the guidelines of the NCEP ATPIII [28]. However, the WC criteria were adapted according to the International Diabetes Federation’s recommendations to consider the unique characteristics of different countries and ethnic groups. Therefore, the WC criteria used in this study were determined using the criteria of the Korean Society for the Study of Obesity [29]. The diagnostic criteria used in this study were based on the criteria employed in previous studies conducted on Koreans [30,31]

To diagnose MetS, three or more of the following five criteria need to be met: WC ≥ 90 cm for men or ≥85 cm for women; fasting blood glucose levels ≥ 100 mg/dL or the use of antidiabetic medication; blood pressure (BP) ≥ 130/85 mmHg or the use of antihypertensive medication; TG ≥ 150 mg/dL; and HDL-C < 40 mg/dL for men or <50 mg/dL for women or the use of anti-dyslipidemic medication.

### 2.3. Anthropometric Measurement and Calculation Method of Indicators

The height of the participants was measured using an extensometer (seca225, Seca, Hamburg, Germany) while they stood upright, and their weight was measured using a digital scale (GL-6000-20, G-tech, Seoul, Republic of Korea). WC was determined by measuring a tape (Seca 200, Seca, Hamburg, Germany) at the midpoint between the lower edge of the last rib and the upper edge of the iliac crest on the participant’s side. To measure body muscle and fat mass, participants underwent a whole-body dual-energy X-ray absorptiometry (DEXA) scan using the equipment (Hologic Discovery-W, Hologic Inc., Bedford, MA, USA).

Height, weight, WC, total muscle mass, and total body fat mass data from the KNHANES dataset were used to calculate the following indicators using the following formulas:

BMI: body weight (kg)/square of height (m^2^) (1)

WHtR: WC (cm)/height (cm)(2)

MFR: total muscle mass (kg)/total fat mass (kg)(3)

MWR: total muscle mass (kg)/WC (cm)(4)

ABSI: WC (m)/[BMI^2/3^ (kg/m^2^) × Height^1/2^ (m)] (5)

### 2.4. Demographic and Health Behavior Factors

Participants’ sex and age were determined using self-reported questionnaires to assess demographic factors. Health behavioral factors were also evaluated using self-reported questionnaires, including current smoking status, alcohol consumption levels, total physical activity levels, average sleep duration, and daily energy intake levels. Smoking status was categorized as current smoker or non-smoker, and alcohol consumption was classified as non-drinking, low drinking, middle drinking, or high drinking based on weekly intake. Total physical activity levels were calculated using the International Physical Activity Questionnaire.

### 2.5. Blood Pressure and Biochemical Measurements

Blood pressure, including systolic and diastolic blood pressure, was measured using a mercury sphygmomanometer (Baumanometer Desk model 0320, BAUM, New York, NY, USA). Blood samples for biochemical analysis were collected from the median cubital vein after an 8 h fasting period. The serum was separated by centrifugation and used for subsequent analyses. Fasting blood glucose, TG, total cholesterol (TC), LDL-C, and HDL-C levels were analyzed using an enzymatic method on an automatic analyzer (Hitachi Automatic Analyzer 7600, Hitachi, Tokyo, Japan). Glycated hemoglobin (HbA1c) levels were measured using high-performance liquid chromatography with an automatic analyzer (HLC-723G7, Tosoh Corporation, Tokyo, Japan).

### 2.6. Statistical Analysis

Categorical variables are presented as frequencies and percentages and were analyzed using the chi-square test. Continuous variables were expressed as means and standard deviations and were compared using an independent *t*-test.

ROC curve analysis was conducted to assess the predictive ability and determine the optimal cutoff values for each anthropometric indicator. The AUC and 95% confidence intervals (CI) were calculated. The cutoff values for each indicator were identified based on the highest Youden Index, calculated as sensitivity + specificity − 1 [32]. The sensitivity, specificity, accuracy, positive predictive value (PPV), and negative predictive value (NPV) were calculated using these cutoff values. The AUCs of each indicator were compared using the nonparametric DeLong test to evaluate their predictive superiority.

Logistic regression analysis was performed to evaluate the association between each indicator and the MetS risk, adjusting for potential covariates. Covariates included age, habitual sleep duration, total calorie intake, alcohol drinking level, total physical activity level, and current smoking status, which were selected for their potential influence on the outcomes. Odds ratios (OR) and 95% Cis were calculated to indicate statistical significance. Statistical analyses, including chi-square tests, independent *t*-tests, and logistic regression analysis, were performed using the SPSS software (version 26.0, IBM Corp., Armonk, NY, USA). ROC curve analysis was conducted using the MedCalc software (version 18.2, MedCalc Software Ltd., Ostend, Belgium). A statistical significance level of 0.05 was applied for all analyses in this study.

## 3. Results

### 3.1. Baseline Demographic and Clinical Characteristics of the Study Participants

In this study, 4911 participants (35.8% of the total) were diagnosed with MetS. The prevalence of MetS was higher in men (2349 [39.9%]) than in women (2562 [32.7%]) (see Table 1). Furthermore, an analysis of the differences between men and women regarding other indicators showed that men had significantly higher values for height, weight, BMI, WC, total muscle mass, MWR, WHtR, MFR, ABSI, TG, TC, LDL-C, fasting blood glucose, HbA1c, systolic blood pressure (SBP), diastolic blood pressure (DBP), total calorie intake, total physical activity level, alcohol consumption level, current smoking status, and MetS components (all *p* < 0.001). In contrast, women demonstrated significantly higher values of fat mass, body fat percentage, and HDL-C levels (all *p* < 0.001). Detailed baseline characteristics and gender-specific differences are summarized in Table 1.

### 3.2. Differences in Measurement Variables Based on the Presence or Absence of Metabolic Syndrome by Gender

Among men with MetS, significantly higher values were observed for various health parameters than among those without MetS. These factors included age, weight, BMI, WC, body fat percentage, fat mass, total muscle mass, WHtR, ABSI, blood lipid levels (TG, TC, and LDL-C), fasting blood glucose, HbA1c, SBP, DBP, average sleep duration, higher prevalence of abdominal obesity, elevated blood glucose, high TG, hypertension, and low HDL-C levels (all *p* < 0.001).

In contrast, men with MetS had significantly lower MWR, MFR, and HDL-C levels. Additionally, alcohol consumption levels, and current smoking status were significantly higher among men with MetS than among those without MetS (all *p* < 0.001). However, there was no significant difference in total calorie intake and physical activity levels between men with and without MetS (all *p* > 0.05).

Similarly, women with MetS had significantly higher age, weight, BMI, WC, body fat percentage, fat mass, total muscle mass, WHtR, ABSI, blood lipid levels (TG, TC, and LDL-C), fasting blood glucose, HbA1c, SBP, DBP, and average sleep duration than those without MetS. Furthermore, women with MetS had a higher prevalence of abdominal obesity, elevated blood glucose levels, high TG levels, hypertension, and low HDL-C levels (all *p* < 0.001). In contrast, women with MetS showed significantly lower MWR, MFR, HDL-C level, alcohol consumption level, and current smoking status (all *p* < 0.001). There was no significant difference in the total calorie intake and total physical activity levels with respect to the prevalence of MetS (all *p* > 0.05). Detailed comparisons of these variables are summarized in Table 2.

### 3.3. Differences in ROC Curve Analysis for Each Indicator

The results of the ROC curve analysis revealed significant differences in the predictive diagnostic power of BMI, MFR, MWR, WHtR, and ABSI.

For men, the AUC values were as follows: BMI, 0.756 (95% CI, 0.744–0.758); WHtR, 0.792 (95% CI, 0.782–0.803); MFR, 0.732 (95% CI, 0.720–0.743); MWR, 0.642 (95% CI, 0.630–0.655); and ABSI, 0.671 (95% CI, 0.659–0.683). The descending order of the AUC values was WHtR, BMI, MFR, ABSI, and MWR, with statistically significant differences observed among the indicators (*p* < 0.001). The analysis also identified the optimal cutoff values, sensitivity, specificity, PPV, NPV, and accuracy for each indicator. The cutoff values for men were as follows: BMI = 24.59 kg/m^2^, WHtR = 0.511 cm/cm, MFR = 3.417 kg/kg, MWR = 0.616 kg/cm, and ABSI = 0.0776 m^11/6^/kg^−2/3^. WHtR demonstrated the highest PPV, NPV, and accuracy among all the indicators.

**Figure 2:**
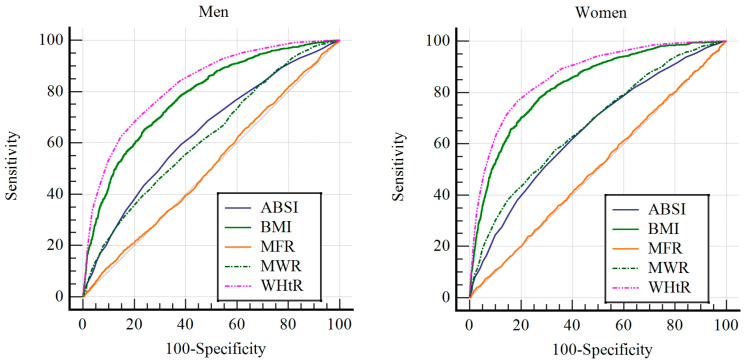
The results of ROC curve analysis for men and women. The left image displays the result of ROC curve analysis for men, while the right image presents the result of ROC curve analysis for women. BMI, body mass index; MWR, muscle mass-to-waist circumference ratio; WHtR, waist-to-height ratio; MFR, muscle-to-fat ratio; ABSI, body shape index.

For women, the AUC values were as follows: BMI, 0.742 (95% CI, 0.732–0.752); WHtR, 0.768 (95% CI, 0.759–0.778); MFR, 0.696 (95% CI, 0.686–0.707); MWR, 0.657 (95% CI, 0.646–0.667); and ABSI, 0.669 (95% CI, 0.659–0.680). Similarly to the findings in men, the AUC values ranked in descending order as WHtR, BMI, MFR, ABSI, and MWR, with statistically significant differences (*p* < 0.001). The analysis also identified the optimal cutoff values, sensitivity, specificity, PPV, NPV, and accuracy for each indicator in women. The cutoff values for women were as follows: BMI = 23.40 kg/m^2^, WHtR = 0.515 cm/cm, MFR = 1.893 kg/kg, MWR = 0.470 kg/cm, and ABSI = 0.0776 m^11/6^/kg^−2/3^. WHtR demonstrated the highest PPV, NPV, and accuracy among the prediction indicators. These findings highlight the superior predictive value of WHtR for both men and women, as summarized in Table 3 and Table 4 and illustrated in Figure 2.

### 3.4. Differences in Prevalence Risk According to the Reference Group for Each Indicator

The logistic regression analysis indicated a significantly higher risk of MetS among participants categorized in the abnormal group (AG) compared to those in the normal group (NG), based on the established cutoff values for each indicator.

In Model 1, which did not adjust for covariates (age, average sleep duration, total calorie intake level, drinking level, total physical activity level, and current smoking status), men in the AG exhibited a significantly higher risk of MetS than those in the NG for all indicators. Specifically, the OR for AG was as follows: BMI, 5.142; WHtR, 7.319; MFR, 4.263; MWR, 2.435; and ABSI, 2.843 (all *p* < 0.001). Similar results were observed in women, with OR of 4.767 for BMI, 6.443 for WHtR, 3.336 for MFR, 2.590 for MWR, and 2.969 for ABSI (all *p* < 0.001).

In Model 2, adjusted for covariates, the results remained consistent. Among men, the AG showed a significantly higher risk of MetS than the NG across all indicators, with adjusted OR as follows: BMI, 5.630; WHtR, 6.793; MFR, 4.514; MWR, 1.823; and ABSI, 2.189 (all *p* < 0.001). Similarly, in women, AG showed significantly higher risks, with adjusted OR of 3.796 for BMI, 4.665 for WHtR, 2.910 for MFR, 1.722 for MWR, and 2.020 for ABSI (all *p* < 0.001).

These findings underscore the significant association between being in the AG and at increased risk of MetS, even after controlling for key covariates. A detailed summary of the logistic regression results is provided in Table 5.

## 4. Discussion

The current study aimed to compare the predictive powers of various anthropometric indicators, including BMI, WHtR, MFR, MWR, and ABSI, for MetS in Korean adults using ROC curve analysis. The primary goal of our investigation was to identify the most effective predictor of MetS in this population.

The findings from the ROC curve analysis provided valuable insights into the accuracy of the cutoff values for predicting diagnostic capability. AUC values below 0.5 are generally considered indicative of poor predictive ability, while values closer to 1 indicate a high diagnostic power [33]. In the present study, all anthropometric indicators, including BMI, WHtR, MFR, MWR, and ABSI, had significant predictive diagnostic abilities for MetS in both men and women, with AUC values exceeding 0.6 and achieving statistical significance. These results consistent with previous studies, which have also confirmed the effectiveness of these indicators in predicting MetS [7,15,16,17,18,22,23]. Specifically, WHtR had the highest AUC values of 0.792 for men and 0.768 for women, indicating the strongest predictive power for MetS among these indicators. Additionally, WHtR demonstrated the highest sensitivity, specificity, PPV, NPV, and accuracy among the indicators. These results are consistent with previous studies that identified WHtR as a superior predictor of both MetS and cardiovascular disease risk compared with other anthropometric indicators [22,23,34,35].

The strong predictive ability of the WHtR can be attributed to its close association with central obesity and visceral fat accumulation, both of which are key features of MetS and significant predictors of cardiometabolic health [36]. Furthermore, a study examining the relationship between various composition indicators and MetS in Korean adults reported similar findings, confirming that the WHtR is the most effective predictor [23]. The ability of the WHtR to incorporate height while reflecting fat distribution makes it particularly valuable in identifying central obesity, a critical component of MetS.

BMI, the most widely used diagnostic tool worldwide, is highly valued in healthcare guidelines for its utility in predicting disease risk [37]. However, despite its widespread use, BMI has a slightly lower predictive power for MetS than WHtR. This reduced predictive ability may be due to the inability of BMI to differentiate between muscle and fat mass and its limited consideration of fat distribution. Previous studies have shown that BMI can sometimes misclassify an individual’s metabolic status, particularly in populations with diverse body compositions [38,39].

MFR and MWR provide additional insights into the role of muscle mass relative to fat mass and waist circumference. While these indicators offer unique perspectives, they are not as effective as the WHtR in predicting MetS. The inclusion of muscle in these ratios reflects their protective role against metabolic diseases. However, previous studies have reported significant differences in muscle mass across different age groups [40,41,42]. In this study, the analysis included participants aged 20–64 years, a broad age range that likely resulted in considerable variation in muscle mass. These age-related variations may have influenced the lower predictive power of the MFR and MWR. To enhance the utility of these measures, future studies should stratify participants by age group in order to better evaluate the predictive power of these indicators within specific age categories.

ABSI is used to assess abdominal obesity and cardiovascular risk, independent of BMI [43]. However, in this study, the AUC of ABSI was lower than that of WHtR. The utility of the ABSI as a better predictor of MetS and mortality than traditional anthropometric measures has been debated in the literature [44,45,46]. Additionally, the small variance in ABSI values complicates the establishment of clinical cutoffs [47]. Moreover, the ABSI’s complex formula makes it less accessible for public use.

The optimal WHtR cutoff values identified in this study for predicting MetS were 0.511 cm/cm for men and 0.515 cm/cm for women. These results align with a previous study among Koreans, which reported optimal WHtR cutoffs of 0.50 cm/cm for men and 0.51 cm/cm for women [48]. Similarly, a meta-analysis involving various ethnic groups reported a cutoff value of 0.5 for WHtR [49].

Logistic regression analysis in this study revealed that participants classified into the AG group for WHtR exhibited the highest risk of MetS in both Model 1 and Model 2. This finding reinforces the role of WHtR as the most effective indicator of central obesity, which is closely associated with MetS. Unlike other indicators, WHtR strongly correlates with visceral fat, a key factor in MetS, making it the most effective index for predicting MetS compared with other anthropometric measures [50,51].

These results suggest that WHtR is a practical and reliable tool for identifying individuals at risk of MetS, particularly in non-clinical or resource-limited settings. Unlike biomedical markers, which require invasive procedures and specialized equipment, WHtR provides a simple, non-invasive, and cost-effective alternative for screening individuals at risk of MetS. Early identification facilitated by WHtR enables timely lifestyle or medical interventions, which could prevent the progression to more severe cardiometabolic conditions [21]. Moreover, WHtR’s simplicity and ease of calculation enhances its utility as a self-monitoring tool, empowering individuals to take an active role in their health management. The utilize of WHtR could encourage proactive health management and preventive behaviors, such as dietary improvements and increased physical activity.

This study had several limitations. First, it employed a cross-sectional design, which hinders the ability to draw causal inferences. Second, as this study was conducted among Koreans, caution is required when generalizing the findings to other ethnic groups. Third, the broad age range of participants may have influenced the ROC curve analysis. Despite these limitations, the present study has several strengths. It identifies non-invasive and easily measurable anthropometric indicators that are most effective in predicting MetS in Korean adults. In addition, the use of raw data obtained from a national institution enhances the credibility of the findings, allowing the conclusions to be extrapolated to the entire Korean population. These strengths highlight the potential of WHtR as a valuable tool for public health initiatives targeting the prevention and management of MetS.

## 5. Conclusions

The present study identified the WHtR as the most accurate and efficient anthropometric indicator for predicting MetS in Korean adults. The WHtR is simple, non-invasive, and cost-effective, making it a highly practical option for assessing MetS and cardiometabolic risk in both clinical and community settings. Additionally, the WHtR provides a more precise measure of fat distribution and central obesity, which are critical factors in the development and progression of MetS. Therefore, the WHtR is the most useful indicator for identifying individuals at risk of developing MetS among Korean adults.

## Figures and Tables

**Table 1:** Characteristic of the study participants.

Variables	Total (*n* = 13,725)	Men (*n* = 5888)	Women (*n* = 7837)	*t*	*p*
Age (years)	42.97 ± 12.13	43.10 ± 12.17	42.87 ± 12.10	1.092	0.275
Height (cm)	163.58 ± 8.73	170.89 ± 6.25	158.09 ± 5.80	122.415	<0.001
Weight (kg)	63.31 ± 11.53	70.65 ± 10.51	57.80 ± 8.89	75.637	<0.001
BMI (kg/m^2^)	23.58 ± 3.35	24.16 ± 3.13	23.14 ± 3.44	18.069	<0.001
WC (cm)	80.32 ± 9.90	84.27 ± 8.84	77.36 ± 9.62	43.604	<0.001
Body fat percentage (%)	28.12 ± 7.58	21.94 ± 5.44	32.76 ± 5.32	−116.490	<0.001
Fat mass (kg)	17.60 ± 5.68	15.66 ± 5.49	19.05 ± 5.38	−36.160	<0.001
Total muscle mass (kg)	42.87 ± 9.38	51.70 ± 6.41	36.23 ± 4.53	157.876	<0.001
MWR (kg/cm)	0.533 ± 0.092	0.616 ± 0.06	0.471 ± 0.05	141.446	<0.001
WHtR (cm/cm)	0.492 ± 0.060	0.494 ± 0.05	0.490 ± 0.06	3.398	0.001
MFR (kg/kg)	2.75 ± 1.33	3.72 ± 1.44	2.03 ± 0.54	85.607	<0.001
ABSI (m^11/6^/kg^−2/3^)	0.076 ± 0.004	0.077 ± 0.004	0.076 ± 0.005	18.404	<0.001
Triglyceride (mg/dL)	130.27 ± 110.15	161.70 ± 137.78	106.65 ± 75.41	27.700	<0.001
Total cholesterol (mg/dL)	186.95 ± 35.43	188.41 ± 35.73	185.85 ± 35.16	4.196	<0.001
LDL-C (mg/dL)	163.96 ± 49.46	174.88 ± 54.28	155.76 ± 43.76	22.155	<0.001
HDL-C (mg/dL)	49.04 ± 11.31	45.87 ± 10.59	51.42 ± 11.26	−29.559	<0.001
Fasting blood glucose (mg/dL)	95.88 ± 21.15	98.50 ± 23.31	93.91 ± 19.13	12.644	<0.001
HbA1c (%)	6.23 ± 1.34	6.35 ± 1.39	6.13 ± 1.29	3.904	<0.001
SBP (mmHg)	116.17 ± 16.15	119.90 ± 14.94	113.37 ± 16.46	24.241	<0.001
DBP (mmHg)	76.70 ± 11.02	80.39 ± 10.75	73.92 ± 10.38	35.390	<0.001
Total calorie intake level (kcal/day)	1994.20 ± 864.91	2429.57 ± 956.83	1705.29 ± 654.06	45.656	<0.001
Average sleep duration (h/day)	6.91 ± 1.69	6.90 ± 2.09	6.91 ± 1.31	−0.169	0.866
Total physical activity level *n* (%)					
low	4142 (30.2)	1475 (25.1)	2667 (34.0)	200.560	<0.001
moderate	5706 (41.6)	2426 (41.2)	3280 (41.9)		
high	3877 (28.2)	1987 (33.7)	1890 (24.1)		
Drinking level *n* (%)					
non-drinking	5659 (41.2)	1272 (21.6)	4387 (56.0)	2741.938	<0.001
low drinking	4723 (34.4)	1998 (33.9)	2725 (34.8)		
middle drinking	2202 (16.0)	1614 (27.4)	588 (7.5)		
high drinking	1141 (8.3)	1004 (17.1)	137 (1.7)		
Current smoker *n* (%)	4539 (33.1)	3851 (65.4)	688 (8.8)	4870.405	<0.001
Metabolic syndrome variables *n* (%)					
abdominal obesity	3176 (23.1)	1517 (25.8)	1659 (21.2)	39.920	<0.001
Elevated blood glucose	6578 (47.9)	3079 (52.3)	3499 (44.6)	78.748	<0.001
High triglyceride	4269 (31.1)	2474 (42.0)	1795 (22.9)	573.161	<0.001
hypertension	7302 (53.2)	3506 (59.5)	3796 (48.4)	166.616	<0.001
low HDL cholesterol	5725 (41.7)	1928 (32.7)	3797 (48.4)	341.072	<0.001
metabolic syndrome	4911 (35.8)	2349 (39.9)	2562 (32.7)	75.926	<0.001

Data are presented as mean ± standard deviation for continuous variables and *n* (%) for categorical variables. BMI, body mass index; WC, waist circumference; MWR, muscle mass-to-waist circumference ratio; WHtR, waist-to-height ratio; MFR, muscle mass-to-fat mass ratio; ABSI, body shape index; LDL-C, low-density lipoprotein cholesterol; HDL-C, high-density lipoprotein cholesterol.

**Table 2:** The differences in variables between men and women with and without MetS.

Variables	Men		*t*	Women		*t*
	Without MetS (*n* = 3539)	With MetS (*n* = 2349)		Without MetS (*n* = 5275)	With MetS (*n* = 2562)	
Age (years)	40.74 ± 12.30	46.66 ± 11.06	−19.213 ***	40.18 ± 11.57	48.41 ± 11.27	−29.812 ***
Height (cm)	171.13 ± 6.22	170.53 ± 6.29	3.613 ***	158.56 ± 5.76	157.11 ± 5.77	10.504 ***
Weight (kg)	67.66 ± 9.35	75.16 ± 10.56	−27.916 ***	55.75 ± 7.64	62.02 ± 9.76	−28.540 ***
BMI (kg/m^2^)	23.08 ± 2.74	25.80 ± 2.97	−35.531 ***	22.18 ± 2.89	25.12 ± 3.64	−35.717 ***
WC (cm)	80.80 ± 7.64	89.49 ± 7.92	−42.147 ***	74.43 ± 8.02	83.40 ± 9.81	−40.242 ***
Body fat percentage (%)	20.21 ± 5.25	24.54 ± 4.61	−33.413 ***	31.57 ± 5.16	35.22 ± 4.78	−30.894 ***
Fat mass (kg)	13.79 ± 4.84	18.49 ± 5.20	−34.946 ***	17.66 ± 4.71	21.91 ± 5.55	−33.279 ***
Total muscle mass (kg)	50.61 ± 6.01	53.33 ± 6.64	−16.007 ***	35.57 ± 4.06	37.60 ± 5.11	−17.591 ***
MWR (kg/cm)	0.628 ± 0.064	0.597 ± 0.060	19.144 ***	0.480 ± 0.051	0.452 ± 0.048	23.554 ***
WHtR (cm/cm)	0.473 ± 0.046	0.525 ± 0.047	−42.349 ***	0.470 ± 0.054	0.532 ± 0.064	−41.467 ***
MFR (kg/kg)	4.14 ± 1.58	3.08 ± 0.871	32.812 ***	2.14 ± 0.56	1.80 ± 0.41	30.294 ***
ABSI (m^11/6^/kg^−2/3^)	0.076 ± 0.004	0.079 ± 0.004	−23.140 ***	0.075 ± 0.004	0.078 ± 0.005	−24.625 ***
Triglyceride (mg/dL)	117.63 ± 88.46	228.11 ± 168.71	−29.189 ***	84.79 ± 47.72	151.67 ± 98.49	−32.563 ***
Total cholesterol (mg/dL)	183.70 ± 32.41	195.52 ± 39.16	−12.132 ***	182.26 ± 32.97	193.25 ± 38.25	−12.464 ***
LDL-C (mg/dL)	157.97 ± 42.62	200.36 ± 59.74	−29.734 ***	144.62 ± 36.24	178.70 ± 48.70	−31.452 ***
HDL-C (mg/dL)	49.25 ± 10.44	40.78 ± 8.58	33.980 ***	54.60 ± 10.98	44.88 ± 8.71	42.458 ***
Fasting blood glucose (mg/dL)	93.12 ± 17.17	106.60 ± 28.45	−20.608 ***	90.26 ± 12.24	101.42 ± 26.98	−19.951 ***
HbA1c (%)	5.88 ± 1.11	6.85 ± 1.47	−12.396 ***	5.63 ± 0.77	7.02 ± 1.52	−18.651 ***
SBP (mmHg)	116.33 ± 13.60	125.29 ± 15.25	−23.030 ***	109.17 ± 13.96	122.04 ± 17.79	−32.146 ***
DBP (mmHg)	77.88 ± 10.08	84.17 ± 10.63	−22.692 ***	71.81 ± 9.52	78.28 ± 10.71	−25.991 ***
Total calorie intake level (kcal/day)	2423.54 ± 948.92	2438.51 ± 968.63	−0.530	1713.40 ± 667.93	1688.61 ± 624.39	1.543
Average sleep duration (h/day)	6.94 ± 2.50	6.85 ± 1.23	1.575	6.95 ± 1.26	6.81 ± 1.40	4.287 ***
Total physical activity level						
low	843 (23.8)	632 (26.9)	20.042 ***	1794 (34.0)	873 (34.1)	0.425
moderate	1424 (40.2)	1002 (42.7)		2219 (42.1)	1.061 (41.4)	
high	1272 (35.9)	715 (30.4)		1262 (23.9)	628 (24.5)	
Drinking level *n* (%)						
non-drinking	787 (22.2)	485 (20.6)	28.330 ***	2801 (53.1)	1586 (61.9)	54.522 ***
low drinking	1266 (35.8)	732 (31.2)		1949 (36.9)	776 (28.5)	
middle drinking	945 (26.7)	669 (28.5)		427 (8.1)	161 (6.3)	
high drinking	541 (15.3)	463 (19.7)		98 (1.9)	39 (1.5)	
Current smoker *n* (%)	2362 (66.7)	1489 (63.4)	7.017 **	517 (9.8)	171 (6.7)	21.049 ***
Metabolic syndrome variables *n* (%)						
High waist circumference	300 (8.5)	1217 (51.8)	1386.081 ***	451 (8.5)	1208 (47.2)	1539.756 ***
High blood glucose	1148 (32.4)	1931 (82.2)	1401.673 ***	1392 (26.4)	2107 (82.2)	2176.669 ***
High triglycerides	665 (18.8)	1809 (77.0)	1964.381 ***	433 (8.2)	1362 (53.2)	1973.444 ***
Hypertension	1447 (40.9)	2059 (87.7)	1281.911 ***	1545 (29.3)	2251 (87.9)	2386.731* **
low HDL-C	541 (15.3)	1387 (59.0)	1227.654 ***	1726 (32.7)	2071 (80.8)	1598.412 ***

Data are presented as mean ± standard deviation for continuous variables and as *n* (%) for categorical variables. BMI, body mass index; WC, waist circumference; MWR, muscle mass-to-waist circumference ratio; WHtR, waist-to-height ratio; MFR, muscle mass-to-fat mass ratio; ABSI, body shape index; LDL-C, low-density lipoprotein cholesterol; HDL-C, high-density lipoprotein cholesterol. ** *p* < 0.01, *** *p* < 0.001.

**Table 3:** The AUC values of each anthropometric indicator.

		AUC	SE	95% CI		Comparison Diagnostics		
				Lower	Upper	All	Men	Women
BMI (a)	All	0.751 ***	0.005	0.744	0.758	d < c < e < a < b	d < e < c < a < b	d < e < c < a < b
	Men	0.756 ***	0.006	0.745	0.767			
	Women	0.742 ***	0.006	0.732	0.752			
WHtR (b)	All	0.778 ***	0.004	0.771	0.785			
	Men	0.792 ***	0.006	0.782	0.803			
	Women	0.768 ***	0.006	0.759	0.778			
MFR (c)	All	0.606 ***	0.005	0.598	0.614			
	Men	0.732 ***	0.006	0.720	0.743			
	Women	0.696 ***	0.006	0.686	0.707			
MWR (d)	All	0.554 ***	0.005	0.545	0.562			
	Men	0.642 ***	0.007	0.630	0.655			
	Women	0.657 ***	0.007	0.646	0.667			
ABSI (e)	All	0.674 ***	0.005	0.666	0.682			
	Men	0.671 ***	0.007	0.659	0.683			
	Women	0.669 ***	0.007	0.659	0.680			

BMI, body mass index; WHtR, waist-to-height ratio; MFR, muscle-to-fat ratio; MWR, muscle mass-to-waist circumference ratio; ABSI, body shape index; AUC, area under the ROC curve; SE, standard error; CI, confidence interval. *** *p* < 0.001.

**Table 4:** The cutoff values and diagnostic powers of BMI, MFR, MWR, WHtR, and ABSI.

Variables		Cutoff	Youden Index	Sensitivity (%)	Specificity (%)	PPV (%)	NPV (%)	Accuracy (%)
BMI (kg/m^2^)	Men	24.59	0.3875	67.0	71.7	61.1	76.6	69.8
	Women	23.40	0.3727	67.6	69.6	51.9	81.5	68.9
WHtR (cm/cm)	Men	0.511	0.4443	63.3	80.9	68.8	76.9	73.9
	Women	0.515	0.4176	61.4	80.2	60.1	81.0	74.1
MFR (kg/kg)	Men	3.417	0.3426	73.0	61.2	55.5	77.3	65.9
	Women	1.893	0.2930	66.4	62.8	46.4	79.4	64.0
MWR (kg/cm)	Men	0.616	0.2182	65.4	56.3	49.8	71.0	60.0
	Women	0.470	0.2333	66.3	56.8	42.7	77.6	60.0
ABSI (m^11/6^/kg^−2/3^)	Men	0.0776	0.2579	62.0	63.5	53.0	71.6	62.9
	Women	0.0776	0.2566	53.2	72.3	48.3	76.1	66.1

BMI, body mass index; WHtR, waist-to-height ratio; MFR, muscle mass-to-fat mass ratio; MWR, muscle mass-to-waist circumference ratio; ABSI, body shape index; PPV, positive predictive value; NPV, negative predictive value.

**Table 5:** The results of the logistic regression analysis based on cutoff values for anthropometric indicators.

Variables		Model 1			Model 2		
		NG	AG		NG	AG	
			OR	95% CI		OR	95% CI
Men	BMI	Reference	5.142 ***	4.593–5.757	Reference	5.630 ***	4.930–6.429
	WHtR	Reference	7.319 ***	6.501–8.241	Reference	6.793 ***	5.929–7.784
	MFR	Reference	4.263 ***	3.806–4.775	Reference	4.514 ***	3.953–5.155
	MWR	Reference	2.435 ***	2.186–2.712	Reference	1.823 ***	1.599–2.079
	ABSI	Reference	2.843 ***	2.552–3.166	Reference	2.189 ***	1.920–2.495
Women	BMI	Reference	4.767 ***	4.308–5.276	Reference	3.796 ***	3.396–4.243
	WHtR	Reference	6.443 ***	5.804–7.152	Reference	4.665 ***	4.151–5.243
	MFR	Reference	3.336 ***	3.021–3.684	Reference	2.910 ***	2.610–3.244
	MWR	Reference	2.590 ***	2.347–2.858	Reference	1.722 ***	1.540–1.926
	ABSI	Reference	2.969 ***	2.692–3.276	Reference	2.020 ***	1.808–2.258

Model 1 was unadjusted, whereas Model 2 was adjusted for age, habitual sleep duration, total calorie intake, drinking level, total physical activity level, and current smoking status. NG, normal group; AG, abnormal group; OR, odds ratio; CI, confidence interval; BMI, body mass index; MWR, muscle mass-to-waist circumference ratio; WHtR, waist circumference-to-height ratio; MFR, muscle mass-to-fat mass ratio; ABSI, body shape index. *** *p* < 0.001.

## Data Availability

KNHANES data used in this study are available at https://knhanes.kdca.go.kr/knhanes/sub03/sub03_02_05.do (assessed on 1 June 2024).
